# Unplanned Mechanical Circulatory Support as Hemodynamic Rescue Worsens Outcomes in Transcatheter Aortic Valve Replacement

**DOI:** 10.3390/jcm15062371

**Published:** 2026-03-20

**Authors:** Michael Keller, Ye In Christopher Kwon, Zachary Gertz, Barbara Lawson, Mohammed Quader, Zubair A. Hashmi

**Affiliations:** 1 Pauley Heart Center, Division of Cardiothoracic Surgery, Department of Surgery, Virginia Commonwealth University School of Medicine, Richmond, VA 23288, USA; 2 Pauley Heart Center, Division of Interventional Cardiology, Department of Medicine, Virginia Commonwealth University School of Medicine, Richmond, VA 23298, USA

**Keywords:** ECMO, TAVR, aortic valve, mechanical circulatory support

## Abstract

**Background/Objectives**: Acute hemodynamic collapse is a rare but deadly complication of transcatheter aortic valve replacement (TAVR) that can require temporary mechanical circulatory support (tMCS). Using a statewide collaborative, we conducted a focused analysis on the incidence and outcomes associated with the use of tMCS during TAVR as hemodynamic rescue. **Methods**: We identified adult patients who underwent TAVR between September 2012 and September 2024 within the statewide collaborative and stratified them based on if tMCS was needed. Baseline patient characteristics and risk factors associated with tMCS use were analyzed as well as the impact of tMCS on outcomes. **Results**: We identified 7735 patients who underwent TAVR. A total of 44 (0.57%) patients required tMCS. Patients requiring tMCS were more likely to have histories that included diabetes, concurrent mitral regurgitation, prior MI, or NYHA class III or IV. These patients also experienced more emergent procedures and were more likely to require inotropic support. Patients experienced significantly worse outcomes following tMCS rescue during TAVR, with 18% requiring conversion to surgical approach (vs. 1%, *p* < 0.001) and 37% of tMCS patients experiencing cardiac arrest, compared to 1% of those who did not need tMCS (*p* < 0.001). Thirty-day mortality was worse for patients requiring tMCS (*p* < 0.001). MCS usage was independently associated with the need for further procedures. **Conclusions**: Unplanned, emergent tMCS during TAVR as hemodynamic rescue represents significant risk of complications and should be utilized judiciously in cases of acute hemodynamic collapse.

## 1. Introduction

Transcatheter aortic valve replacement (TAVR) is commonly used as an alternative option for treatment in patients with aortic stenosis who are at elevated risk of morbidity and mortality if undergoing surgical aortic valve replacement (SAVR). Acute hemodynamic collapse is a rare but deadly complication of TAVR, occurring in approximately 1.5–4.5% of procedures [1,2,3]. Prompt initiation of temporary mechanical circulatory support (tMCS) is indicated for procedure completion and avoidance of intraoperative mortality in cases of acute hemodynamic collapse.

However, tMCS usage during TAVR is not without its risks. It has been reported to be associated with increased mortality rates, longer length of stay, and increased risks of bleeding, renal failure, post-operative myocardial infarctions (MI) and strokes [3,4]. Despite these risks, tMCS rescue is necessary to prevent intraprocedural mortality. Prior studies include case reports and single-center experiences with tMCS ECMO to analyze comorbidity burden and timing of procedures [4,5]. We utilized a statewide collaborative to conduct a focused analysis on the incidence and outcomes associated with the use of tMCS for hemodynamic rescue during TAVR.

## 2. Materials and Methods

**Figure 1:**
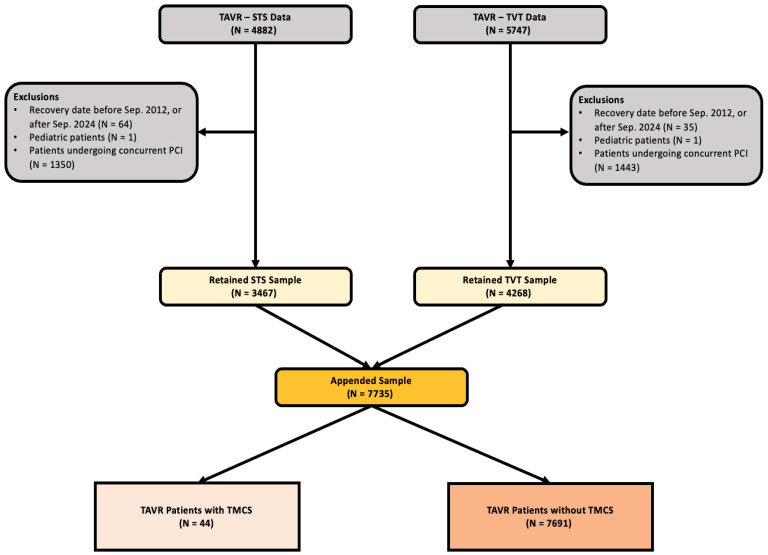
Patient flow diagram.

This was a retrospective cohort study of a statewide, prospectively collected database of cardiac surgery patients (utilizing Society for Thoracic Surgeons (STS) and Transcatheter Valve Therapy registries). We identified all adult patients who underwent TAVR between September 2012 and September 2024 as seen in Figure 1. Patients were excluded if they were under the age of 18 or were undergoing concurrent percutaneous coronary intervention (PCI). They were further stratified based on intraoperative requirements of tMCS, including intra-aortic balloon pump (IABP), extracorporeal membrane oxygenation (ECMO), and percutaneous ventricular assist devices (pVAD).

Patient characteristics and risk factors associated with tMCS use were analyzed with mean ± standard deviation (SD) and frequencies with percentages for continuous and categorical variables respectively. Pearson’s χ^2^, Fisher’s exact test and the Kruskal–Wallis test were used as appropriate. Thirty-day mortality was analyzed for patients included in the Society of Thoracic Surgeons database using the Kaplan–Meier method. The impact of tMCS on complications following TAVR was analyzed via multivariate logistic regression models. The impact on mortality was analyzed using multivariate cox-regression. This study utilized deidentified data provided by the statewide collaborative. As no protected health information or patient identifiers were included in the dataset, this study was determined to be exempt from Institutional Review Board review at Virginia Commonwealth University in accordance with 45 CFR 46.104(d)(4). 2.

## 3. Results

### 3.1. Patient Characteristics

A total of 7735 patients underwent TAVR, of which 44 (0.57%) required unplanned intraoperative tMCS. Patients requiring tMCS were more likely to be female (71%, *p* = 0.002) and to be White (68%, *p* = 0.004, Table 1). Those who required tMCS were more likely to classify as New York Heart Association Class III or IV (71% vs. 51%, *p* = 0.009), have concurrent moderate or severe mitral valve regurgitation (34% vs. 18%, *p* = 0.008), diabetes (52% vs. 37%, *p* = 0.04), or a prior myocardial infarction (21% vs. 11%, *p* = 0.03). Patients with need for tMCS had higher rates of inotrope requirement within 48 h of TAVR (14% vs. 3%, *p* < 0.001). Procedures requiring tMCS were more likely to be emergent (7% vs. 0.2%), salvage (5% vs. 0%), or urgent (32% vs. 9%, all *p* < 0.001). These procedures were more likely to have concurrent pacemaker implantation (36% vs. 4%) and require general anesthesia (57% vs. 38%, all *p* < 0.001). Patients who required tMCS were less likely to have undergone a transfemoral approach (82% vs. 95%, *p* < 0.01).

Patients requiring tMCS demonstrated differences in preoperative aortic valve hemodynamics compared to those not requiring tMCS. Aortic valve area was significantly smaller in the tMCS group (0.70 [0.55–0.80] vs. 0.72 [0.57–0.78] cm^2^, *p* = 0.0021), and aortic valve peak velocity was significantly lower (3.70 [3.20–4.20] vs. 4.10 [3.70–4.50] m/s, *p* = 0.0033). Aortic valve mean gradient trended lower in the tMCS group but did not reach statistical significance (39.5 [29.0–46.8] vs. 41.0 [33.8–48.7] mmHg, *p* = 0.1327).

Among the 44 patients requiring tMCS, ECMO was the most frequently utilized modality (*n* = 18, 40.9%), followed by IABP (*n* = 17, 38.6%) and pVAD (*n* = 9, 20.5%). IABP use was concentrated in the earlier study period (2012–2014), while ECMO was utilized consistently throughout, and pVAD adoption increased in later years. A percutaneous transfemoral approach was used for valve/sheath access in the majority of tMCS patients (*n* = 36, 81.8%), with surgical cutdown (*n* = 4, 9.1%), mini sternotomy (*n* = 2, 4.5%), and mini thoracotomy (*n* = 2, 4.5%) comprising the remaining cases. Regarding timing, no patients had tMCS in place at the start of the procedure, confirming the unplanned nature of tMCS in this cohort. The majority of tMCS was initiated after intervention had begun (*n* = 28, 63.6%), with smaller proportions inserted during the procedure but prior to valve intervention (*n* = 8, 18.2%) or in the immediate post-procedural period (*n* = 8, 18.2%).

### 3.2. Risk Factors for tMCS Requirements

In multivariate regression analysis, operative characteristics were predictive of need for tMCS rescue (Table 2). Salvage TAVR procedures had an 18-fold higher chance of needing tMCS rescue (adjusted odds ratio (aOR) 18.86, 95% confidence intervals (CI) 4.99–69.2, *p* = 0.005). Concurrent pacemaker implantation was also associated with higher likelihood of needing tMCS rescue (aOR 28.55, 95% CI 6.31–129.1, *p* < 0.001). No baseline demographics, laboratory values, or comorbid conditions were associated with tMCS requirements.

### 3.3. Mortality for Patients Requiring tMCS

**Figure 2:**
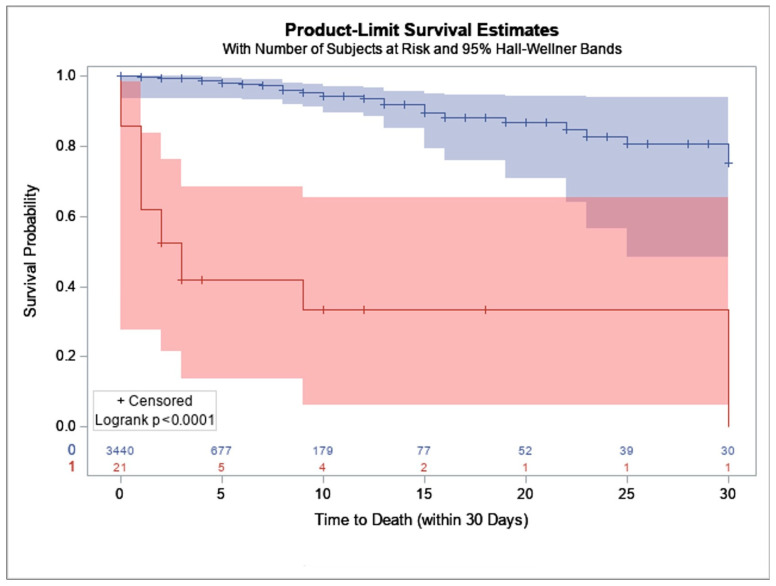
Kaplan–Meier plot of 30-day mortality. tMCS rescue represented in red, no tMCS represented in blue.

In the STS dataset, 21 patients required tMCS and 3440 did not. Of the 21 patients requiring tMCS represented in the STS database, 16 of them died within 5 days of their initial TAVR procedure; and at 30 days only one patient was still living. Thirty-day mortality was significantly increased in patients who required tMCS (*p* < 0.001, Figure 2). On multivariate analysis, need for tMCS was associated with higher risk of mortality (adjusted hazard ratio (aHR) 6.67, 95% CI 1.83–14.39, *p* < 0.001).

Comorbidities associated with increased risk of mortality following tMCS rescue included mitral stenosis (aHR 2.76, 95% CI 1.09–7.03, *p* = 0.033, Table 3) and prior coronary artery bypass surgery (aHR 4.11, 95% CI 1.45–11.68, *p* = 0.008). Operative characteristics increasing mortality included concurrent pacemaker implantation (aHR 6.74, 95% CI 2.39–18.96, *p* < 0.001) and use of general anesthesia (aHR 2.77, 95% CI 1.17–6.56, *p* = 0.021). Use of beta blockers within 24 h of TAVR was protective against mortality following tMCS (aHR 0.29, 95% CI 0.12–0.66, *p* = 0.0035).

### 3.4. Adverse Outcomes of Patients Requiring tMCS

Patients who required tMCS during TAVR had significantly worse outcomes when compared to patients not requiring tMCS. These patients were more likely to require conversion to a surgical approach (18% vs. 1%), suffer cardiac arrest (37% vs. 1%), have significant bleeding (32% vs. 2.3%), and experience aortic dissection (5% vs. 0.2%) or other vascular complications (23% vs. 3%, all *p* < 0.001, Table 4). Following their TAVR procedure, patients with tMCS experienced higher rates of post-operative stroke (9% vs. 2%, *p* < 0.001) and renal failure requiring dialysis (2% vs. 0.4%, *p* < 0.048). Furthermore, they had higher rates of unplanned coronary artery intervention (9% vs. 0.3%) and reoperation due to valvular dysfunction (7% vs. 0.2%, all *p* < 0.001).

Adjusted analyses demonstrated similar directionality to unadjusted models. After logistic regression modeling, tMCS was independently associated with both intraprocedural and post-procedural complications (Table 5). Patients who required tMCS support had a higher likelihood of needing to convert to an open operation (aOR 21.71, 95% CI 9.8–48.2, *p* < 0.001). Rescue mechanical circulatory support was associated with higher rates of cardiac arrest (aOR 55.67, 95% CI 29.27–105.9), bleeding (aOR 55.67, 95% CI 29.27–105.9), aortic dissection (aOR 28.13, 95% CI 6.16–128.51) and other vascular complications (aOR 8.31, 95% CI 4.06–16.99, all *p* < 0.001). Need for tMCS was also associated with increased risk of stroke in the post-operative period (aOR 6.31, 95% CI 2.22–17.91), reoperations for valvular dysfunction (aOR 43.22, 95% CI 11.87–157.37), and unplanned coronary artery interventions (aOR 38.37, 95% CI 12.55–117.28).

## 4. Discussion

Across a 12-year retrospective study, we found that emergent tMCS used for hemodynamic rescue during TAVR procedure was associated with intra- and post-procedural complications. Additionally, we found a heterogenous cohort of patients with increased comorbidity burden prior to procedure that was more likely to require tMCS rescue. Our study found lower statewide usage of tMCS when compared to both single-center and national studies [1,2,4]. However, patients supported with tMCS were similarly high risk pre-operatively, with greater comorbidity burden than patients who did not require tMCS. Following intraprocedural rescue with temporary mechanical circulatory support, patients requiring tMCS rescue experienced significantly increased mortality and adverse effects following TAVR. While the original TAVR procedure was indicated for patients with severe aortic stenosis considered at high risk for surgical repair or replacement, it is now indicated across all risk categories, and assessing patients at high risk for intraprocedural complications is consequently of utmost importance.

Prior studies have also documented a higher comorbidity burden experienced by patients who go on to require tMCS rescue during TAVR procedures. Shou et al. report significantly higher Elixhauser comorbidity index among patients who require tMCS, and Banga et al. report higher EuroSCORE in patients who required ECMO for TAVR [3,4]. The Elixhauser comorbidity index was designed for application across wide ranges of administrative data and includes congestive heart failure, valvular disease, diabetes, mental health disorders and drug abuse among its 30 factors [6]. EuroSCORE has been validated as a surgical risk calculator and includes similar factors [7].

Similar to the studies mentioned before, we also report an increased risk of needing tMCS during TAVR in patients who have higher comorbidity burden or more acute presentations. We report higher rates of NYHA class III and IV heart failure represented amongst patients who require tMCS during TAVR. In patients already experiencing symptomatic heart failure, rapid ventricular pacing (RVP) during valve deployment and cardiac stunning during the TAVR procedure can preclude acute cardiovascular collapse [8,9,10]. Despite higher rates of NYHA class III and IV heart failure, patients who require tMCS have a mean LVEF that is not significantly different from those not needing tMCS, suggesting that symptoms of heart failure rather than hemodynamic measures are more related to tMCS need.

Our data shows higher rates of prior MI and diabetes in patients who require tMCS; however, neither were independently associated with the need for tMCS rescue. Patients with a prior MI may have residual scarring and impaired function [11]. While this may impact long term left ventricular remodeling and LVEF improvement, it has not been shown to independently impact mortality after TAVR. Despite this, persistent effects of prior MI may impact patients’ physiology and their need for tMCS. Patients requiring tMCS were also shown to have higher rates of diabetes, a comorbidity present in many patients needing tMCS and itself associated with higher risk of in-hospital mortality [12]. While diabetes may not directly impact patients’ need for tMCS, it is often a signal of underlying pathophysiology that can lead to adverse outcomes.

Patients who ultimately require tMCS are also more likely to be in extremis upon presentation. Shou et al. reported that nonelective admission was associated with higher rates of need for tMCS utilization, whereas elective admission independently predicted a reduced need for tMCS [4]. Similarly, elective procedures in our cohort were associated with a lower likelihood of needing tMCS, while salvage, urgent, and emergent procedures were significantly associated with increased use of mechanical support. Salvage procedures were an independent predictor of need for tMCS. Along with procedural status, we report that tMCS patients were more likely to have required inotropic support in the 48 h prior to TAVR. Particularly in patients with aortic stenosis, the use of inotropic agents for increased mean arterial pressure must be weighed against the potential for worsening of the aortic valve pressure gradient [13]. When used in the period prior to definitive therapy, this could be a necessary tradeoff but does present a patient who may not be fully optimized prior to TAVR.

Our data showed that patients requiring tMCS presented with smaller aortic valve areas and lower peak velocities. In a cohort of patients requiring ECMO support during TAVR procedure, Seco et al. report similar findings of smaller aortic valve area and mean aortic gradient [14]. While our patients present with a trend toward decreased gradient, combined with smaller valve area and lower peak velocities, these patients may represent a “low-flow, low-gradient” state [12,15]. With preserved ejection fraction, this subset of patients has been described as paradoxical low-flow, low-gradient (PLFLG) and is associated with an underestimation of aortic stenosis severity [12]. PLFLG patients have higher mortality following TAVR and commonly have a higher comorbidity burden [12,16,17]. To evaluate low-flow, low-gradient patients, Delgado et al. discuss the importance of dobutamine stress echocardiography to determine severity of aortic stenosis and aortic valve calcium scoring to characterize the valvular anatomy [18]. When coupled with urgent or emergent presentations, full evaluation with dobutamine stress testing may not be feasible, and preprocedural discussion for low-flow, low-gradient patients should address the increased risk of requiring tMCS rescue.

We report increased risk of tMCS need in patients with severe mitral regurgitation who undergo TAVR and an independent association between mitral stenosis and increased mortality. Prior studies highlight the complex interplay between aortic and mitral valve pathologies. Previously, Bhogal et al. presented a detailed list of factors to be considered when deciding between TAVR and SAVR [19]. Characteristics that have unclear evidence for one procedure compared to the other include severe mitral stenosis, regurgitation, and tricuspid regurgitation. In combination with the sudden afterload reduction seen after valve deployment, pre-existing mitral regurgitation can be exacerbated. In some cases this can lead to dynamic left ventricular outflow tract obstruction, either by wire-related complications or systolic anterior motion of the mitral valve [20]. The resulting hypotension can lead to a need for tMCS if refractory to medication. With regard to mitral stenosis, patients with severe stenosis experience increased adverse outcomes following TAVR, including increased heart failure hospitalizations and mortality, and are at increased risk of post-TAVR aortic paravalvular leak [21,22].

Patients requiring tMCS for hemodynamic rescue during TAVR were found to have primarily with IABP and ECMO; however, over 20% of patients were supported with pVADS. While stenotic valves were originally considered a contraindication to pVAD support, studies have shown that pVAD support is both feasible and safe for use despite the presence of aortic valve stenosis [23,24].

Our data shows that need for tMCS was associated with significantly increased morbidity and higher likelihood of subsequent interventions, including unplanned coronary artery interventions and vascular repair. These patients were also at higher risk of aortic dissection, persistent valvular dysfunction, and post-operative bleeding, stroke, and renal failure requiring dialysis. Ultimately, we report significantly increased risk of 30-day mortality for patients who require tMCS during TAVR and that intraprocedural initiation of tMCS is independently associated with increased risk of mortality. When initiated emergently, ECMO, a form of tMCS, has been shown to increase adverse events and mortality across a number of procedures, including after cardiopulmonary bypass and PCI [25,26]. It is important to note, however, that a common indication for emergent tMCS is refractory cardiac arrest, which itself portends to worsened outcomes. Banga et al. compared ECMO initiated during procedure to ECMO initiated prior to procedure for planned high-risk TAVR and found that while rescue ECMO had increased mortality compared to prophylactic ECMO, both cohorts experienced increased mortality when compared to no-ECMO cohorts [3]. Our findings include a larger range of tMCS modalities, including ECMO, IABP and pVAD, but support the findings of Banga et al., as tMCS was associated with increased risk of mortality.

It becomes difficult to delineate between the physiological or procedural components of increased morbidity. Patients in our cohort requiring tMCS rescue are higher-risk patients prior to their TAVR procedure, which in and of itself lends towards post-operative stroke and renal failure [27,28]. Pre-existing mitral valve dysfunction has been shown to complicate TAVR and lead to decreased survival and increased hospitalizations due to heart failure post-TAVR [29]. Ali et al. describe common complications associated with mechanical circulatory support usage, including increased risk of vascular complications due to indwelling devices, major bleeding associated with therapeutic anticoagulation, and stroke either from anticoagulation or thrombus disruption [30].

Limitations of this study are largely related to the retrospective nature of our cohort and the relatively small number of patients who required tMCS. Granular clinical data, such as preoperative hemodynamic data, and long-term follow-up were not included in the database. Furthermore, we were unable to stratify patients based on pre-operative risk scores such as the EuroSCORE II or other TAVR-specific risk stratification calculators. Secondly, caution must be used due to the disparity in cohort sizes between tMCS and no-tMCS cohorts. The number of patients requiring tMCS was relatively small, reflecting the infrequent need for escalation to mechanical circulatory support. However, directionality was maintained on both adjusted and unadjusted analyses, and, in this case, smaller cohort sizes likely lead to limited precision rather than a lack of association. Additionally, sample size led to an inability to stratify device choices. Differences in clinical scenarios affect choice of device, as do institutional practice patterns, leading to issues with generalizing these findings with respect to devices. Further research is warranted to best define patients most at risk of needing emergent intraprocedural tMCS and to help prevent adverse outcomes.

Despite the inherent risks associated with intraprocedural tMCS rescue, it is important to recognize the emergent nature of this intervention. Acute hemodynamic collapse is a fatal complication of TAVR without intervention and must be weighed with the risk of further procedures, need for dialysis, or unplanned conversion to an operative approach. Given the risk factors for tMCS need, namely inotrope usage prior to TAVR, mitral valve regurgitation and symptomatic heart failure, pre-operative discussions should include the unlikely but very real possibility of need for tMCS and the associated increased morbidity and mortality.

## 5. Conclusions

With the growing usage of TAVR across wide-ranging patient populations, preprocedural risk stratification is of utmost importance. Our study presents important information on the usage of tMCS for hemodynamic collapse to guide preparedness and patient counseling prior to procedure. Patients with elevated risk profiles, including symptomatic heart failure, prior myocardial infarctions, concomitant mitral valve pathologies, and diabetes, are at higher risk of needing tMCS. Additionally, recent inotropic usage, as well as acuity of the procedure, lead to higher risk. This risk also translates into more adverse outcomes post-intervention and longer length of stay. Furthermore, tMCS is associated with persistent valvular dysfunction post-procedure, increased need for coronary artery interventions, increased risk of post-operative bleeding, stroke, and dialysis, as well as increased 30-day mortality.

## Figures and Tables

**Table 1:** Demographics and baseline clinical characteristics of TAVR patients who did not require versus did require temporary mechanical circulatory support.

	Overall	Not Requiring tMCS	Requiring tMCS	
Variables	(*n* = 7735)	(*n* = 7691)	(*n* = 44)	*p* Value
Age	78 [72–84]	78 [72–84]	74 [69–88]	0.3483
Sex				0.0024
Male	4051 (52.3%)	4038 (52.5%)	13 (29.6%)	
Female	3683 (47.6%)	3652 (47.5%)	31 (70.5%)	
Race				0.0040
White	6516 (84.2%)	6486 (84.3%)	30 (68.2%)	
Black	828 (10.7%)	816 (10.6%)	12 (27.3%)	
Hispanic/Latino	95 (1.2%)	95 (1.2%)	0 (0%)	
Other	296 (3.8%)	294 (3.8%)	2 (4.6%)	
BMI	28.2 [24.5–33]	28.2 [24.5–33]	26.7 [22.8–30.2]	0.2268
Preop Labs				
Creatinine (mg/dL)	1 [0.8–1.3]	1 [0.8–1.3]	1 [0.8–1.5]	0.6669
Total Bilirubin (mg/dL)	0.5 [0.4–0.8]	0.5 [0.4–0.8]	0.6 [0.4–0.9]	0.6210
Albumin (g/dL)	4 [3.6–4.3]	4 [3.6–4.3]	3.9 [3.5–4.2]	0.3974
Hemoglobin (g/dL)	12.5 [11.2–13.6]	12.5 [11.2–13.6]	11.7 [9.7–13.3]	0.3124
Platelet count ×10^9^	207 [169–254]	207 [169–253]	199 [147–270]	0.6459
Preop Hemodynamics				
Aortic Valve Area (cm^2^)	0.72 [0.60–0.90]	0.72 [0.57–0.78]	0.70 [0.55–0.80]	**0.0021**
Aortic Valve Mean Gradient (mmHg)	41.0 [34.0–50.0]	41.0 [33.8–48.7]	39.5 [29.0–46.8]	0.1327
Aortic Valve Peak Velocity (m/s)	4.00 [3.70–4.40]	4.10 [3.70–4.50]	3.70 [3.20–4.20]	**0.0033**
Ejection Fraction (%)	60 [55–63]	60 [55–63]	55 [30–65]	0.2463
Comorbidities				
NYHA Heart Disease Class III or IV	3936 (50.9%)	3905 (50.8%)	31 (70.5%)	**0.0092**
Moderate-Severe Mitral Valve Regurgitation	1433 (18.5)	1418 (18.4%)	15 (34.1%)	**0.0077**
Mitral Stenosis	763 (9.9%)	756 (9.8%)	7 (15.9%)	0.1775
Moderate–Severe Tricuspid Regurgitation	1147 (14.8%)	1138 (14.8%)	9 (20.5%)	0.2923
Moderate–Severe Lung Disease	1038 (13.4%)	1032 (13.4%)	6 (13.6%)	0.9662
History of Smoking	4012 (51.9%)	3994 (51.9%)	18 (40.9%)	0.1446
Hypertension	6881 (89.0%)	6841 (89%)	40 (90.9%)	0.6790
Diabetes Mellitus	2901 (37.5%)	2878 (37.4%)	23 (52.3%)	**0.0424**
Prior Myocardial Infarction	824 (10.7%)	815 (10.6%)	9 (20.5%)	**0.0346**
Prior Coronary Artery Bypass	911 (11.8%)	907 (11.8%)	4 (9.1%)	0.5793
Prior Balloon Aortic Valvuloplasty	921 (11.9%)	913 (11.9%)	8 (18.2%)	0.1975
Prior Surgical Aortic Valve Procedure	471 (6.1%)	468 (6.1%)	3 (6.8%)	0.8393
Prior Transcatheter Aortic Valve Replacement	60 (0.8%)	60 (0.8%)	0 (0%)	0.5564
Coronary Artery Disease	3447 (44.6%)	3430 (44.6%)	17 (38.6%)	0.4276
Previous Cerebrovascular Accident	878 (11.4%)	875 (11.4%)	3 (6.8%)	0.3418
Peripheral Arterial Disease	1568 (20.3%)	1557 (20.2%)	11 (25%)	0.4340
Preoperative Dialysis	279 (3.6%)	275 (3.6%)	4 (9.1%)	0.0504
Cardiac Arrhythmia	2646 (34.2%)	2635 (34.3%)	11 (25%)	0.1966
Preoperative Medications				
ACE inhibitor and Angiotensin-Receptor Blocker	1126 (14.6%)	1122 (14.6%)	4 (9.1%)	0.3025
Beta Blockers w/in 24 h	1744 (22.5%)	1732 (22.5%)	12 (27.3%)	0.4519
Aspirin	2066 (26.7%)	2051 (26.7%)	15 (34.1%)	0.2671
Home Oxygen	471 (6.1%)	466 (6.1%)	5 (11.4%)	0.1423
Anticoagulants w/in 48 h	279 (3.6%)	275 (3.6%)	4 (9.1%)	0.0504
Inotropes w/in 48 h	267 (3.5%)	261 (3.4%)	6 (13.6%)	**0.0002**
Immunocompromise	370 (4.8%)	368 (4.8%)	2 (4.6%)	0.9409
Operative Characteristic				
Status of operation				**<0.0001**
Elective	7009 (90.6%)	6984 (90.8%)	25 (56.8%)	
Emergency	19 (0.2%)	16 (0.2%)	3 (6.8%)	
Salvage	5 (0%)	3 (0%)	2 (4.6%)	
Urgent	701 (9.1%)	687 (8.9%)	14 (31.8%)	
Transfemoral	7357 (95.1%)	7321 (95.2%)	36 (81.8%)	**<0.0001**
MCS Modality				
IABP	17	-	17 (38.6%)	**-**
ECMO	18	-	18 (40.9%)	**-**
pVAD	9	-	9 (20.5%)	**-**
Valve/Sheath Access Site				
Percutaneous	36	-	36 (81.8%)	**-**
Surgical Cutdown	4	-	4 (9.1%)	**-**
Mini Sternotomy	2	-	2 (4.5%)	**-**
Mini Thoracotomy	2	-	2 (4.5%)	**-**
Timing of MCS Initiation				
In place at start of TAVR	0	-	0 (0%)	**-**
Inserted prior to TAVR	8	-	8 (18.2%)	**-**
Inserted after TAVR begun	28	-	28 (63.6%)	**-**
Post-TAVR	8	-	8 (18.2%)	**-**
Concurrent Pacemaker Implantation	303 (3.9%)	287 (3.7%)	16 (36.4%)	**<0.0001**
General Anesthesia	2914 (37.7%)	2889 (37.6%)	25 (56.8%)	**0.0086**
Procedural Sedation	3395 (43.9%)	3388 (44.1%)	7 (15.9%)	**0.0002**

Data are presented as median [interquartile range] for continuous variables and percentages for dichotomous variables; NYHA—New York Heart Association, - is for data only collected for one cohort

**Table 2:** Predictors of temporary mechanical circulatory support rescue among TAVR patients.

Variables	Adjusted Odds Ratio	95% Confidence Interval	*p* Value
Age	0.96	0.91–1.02	0.2172
Sex			
Male	Reference		
Female	3.33	0.84–13.21	0.0876
Race			
White	Reference		
Black	1.71	0.41–7.07	0.4600
Other	1.92	0.2–18.62	0.5756
BMI	0.99	0.91–1.08	0.8577
Preop Labs			
Creatinine (mg/dL)	1.38	0.94–2.03	0.1000
Total Bilirubin (mg/dL)	0.67	0.11–4.02	0.6594
Albumin (g/dL)	1.36	0.52–3.57	0.5341
Hemoglobin (g/dL)	0.95	0.67–1.34	0.7496
Platelet count ×10^9^	1.00	0.99–1.01	0.6554
Preop Hemodynamics			
Ejection Fraction (%)	0.97	0.93–1.01	0.1973
Comorbidities			
NYHA Heart Disease Class III or IV	2.03	0.48–8.63	0.3384
Moderate–Severe Mitral Valve Regurgitation	0.98	0.23–4.26	0.9807
Mitral Stenosis	1.44	0.28–7.37	0.6582
Moderate–Severe Tricuspid Regurgitation	0.28	0.04–1.79	0.1784
Moderate–Severe Lung Disease	0.20	0.02–1.91	0.1613
History of Smoking	0.51	0.15–1.72	0.2750
Hypertension	0.47	0.08–2.89	0.4123
Diabetes Mellitus	1.58	0.46–5.49	0.4687
Prior MI	2.98	0.8–11.14	0.1043
Prior CAB	0.51	0.05–5.14	0.5688
Prior Balloon Aortic Valvuloplasty	2.76	0.76–10.08	0.1245
Prior Surgical Aortic Valve Procedure	1.07	0.15–7.78	0.9450
CAD	2.25	0.44–11.46	0.3272
Peripheral Arterial Disease	1.42	0.37–5.47	0.6099
Preoperative Dialysis	0.83	0.04–16.03	0.9040
Cardiac Arrhythmia	0.85	0.24–3.08	0.8088
Preoperative Medications			
ACEi/ARB	0.51	0.07–3.49	0.4918
Beta Blockers w/in 24 h	1.89	0.41–8.73	0.4148
Aspirin	1.61	0.35–7.42	0.5406
Home Oxygen	1.15	0.12–10.96	0.9046
Inotropes w/in 48 h	0.79	0.1–6.02	0.8188
Operative Characteristics			
Status of Operation			
Urgent	Reference		
Elective	0.34	0.08–1.56	0.1653
Emergency	10.44	0.26–41.65	0.2115
Salvage	18.86	4.99–69.15	0.0047
Concurrent Pacemaker Implantation	28.55	6.31–129.1	<0.0001
General Anesthesia	0.89	0.13–6.27	0.9067
Procedural Sedation	0.83	0.15–4.77	0.8378

Data are presented as median [interquartile range] for continuous variables and percentages for dichotomous variables. NYHA: New York Heart Association, MI: myocardial infarction, CAB: coronary artery bypass, TAVR: trans-catheter aortic valve replacement, CAD: coronary artery disease.

**Table 3:** Multivariate cox hazard model of mortality for patients who did and did not require tMCS.

Variables	Adjusted Hazard Ratio	95% Confidence Interval	*p* Value
TMCS			
Not requiring tMCS	Reference		
Requiring tMCS	6.67	1.83–14.39	<0.0001
Age	1.03	0.98–1.08	0.2304
Sex			
Male			
Female	1.81	0.79–4.18	0.1625
Race			
White	Reference		
Black	0.94	0.26–3.46	0.9278
H/L	NA	NA	NA
Other	0.44	0.04–4.55	0.4883
BMI	1.00	0.99–1.01	0.9128
Preop Labs			
Creatinine (mg/dL)	1.13	0.86–1.51	0.3825
Total Bilirubin (mg/dL)	0.96	0.8–1.16	0.6942
Albumin (g/dL)	0.73	0.35–1.53	0.4033
Hemoglobin (g/dL)	0.87	0.7–1.08	0.1966
Platelet count ×10^9^	1.00	0.99–1	0.5524
Preop Hemodynamics			
Ejection Fraction (%)	1.02	0.98–1.07	0.3405
Comorbidities			
NYHA Heart Disease Class III or IV	1.02	0.36–2.9	0.9734
Moderate-Severe Mitral Valve Regurgitation	1.07	0.47–2.42	0.8805
Mitral Stenosis	2.76	1.09–7.03	**0.0330**
Moderate–Severe Tricuspid Regurgitation	0.41	0.11–1.51	0.1812
Moderate–Severe Lung Disease	1.54	0.61–3.85	0.3580
History of Smoking	0.50	0.21–1.21	0.1254
Hypertension	NA	NA	NA
Diabetes Mellitus	0.59	0.25–1.41	0.2370
Prior Myocardial Infarction	1.10	0.29–4.15	0.8926
Prior Coronary Artery Bypass	4.11	1.45–11.68	**0.0080**
Prior Balloon Aortic Valvuloplasty	1.43	0.58–3.53	0.4389
Prior Surgical Aortic Valve Procedure	1.11	0.14–8.9	0.9202
Prior Transcatheter Aortic Valve Replacement	0.00	0–0	**<0.0001**
Coronary Artery Disease	0.77	0.17–3.39	0.7253
Previous Cerebrovascular Accident	0.44	0.07–2.87	0.3902
Peripheral Arterial Disease	1.16	0.59–2.29	0.6744
Preoperative Dialysis	0.77	0.11–5.38	0.7890
Cardiac Arrythmia	1.31	0.65–2.63	0.4571
Preoperative Medications			
ACE-inhibitor/Angiotensin-Receptor Blocker	0.53	0.17–1.6	0.2576
Beta Blockers w/in 24 h	0.29	0.12–0.66	**0.0035**
Aspirin	0.59	0.26–1.34	0.2098
Home Oxygen	0.64	0.22–1.86	0.4060
Anticoagulants w/in 48 h	NA	NA	NA
Inotropes w/in 48 h	NA	NA	NA
Immunocompromise	0.90	0.22–3.67	0.8853
Operative Characteristics			
Status of operation			
Urgent	Reference		
Elective	2.73	0.54–13.91	0.2258
Emergency	6.33	0.44–92.08	0.1768
Salvage	0.00	0–0	**<0.0001**
Transfemoral	0.43	0.17–1.07	0.0680
Concurrent Pacemaker Implantation	6.74	2.39–18.96	**0.0003**
General Anesthesia	2.77	1.17–6.56	**0.0206**
Procedural Sedation	0.88	0.29–2.7	0.8215

Data are presented as median [interquartile range] for continuous variables and percentages for dichotomous variables. NYHA: New York Heart Association. Results reported as NA when population size prohibits data analysis.

**Table 4:** Adverse outcomes for patients who did not require versus did require tMCS.

	Overall	No tMCS	tMCS	
Variables	(*n* = 7735)	(*n* = 7691)	(*n* = 44)	*p* Value
Conversion of Operative Approach	86	78 (1%)	8 (18.2%)	<0.0001
Aortic Dissection	15	13 (0.2%)	2 (4.6%)	<0.0001
Vascular Complications	273	263 (3.4%)	10 (22.7%)	<0.0001
Cardiac Arrest	103	86 (1.1%)	17 (38.6%)	<0.0001
Bleeding	194	180 (2.3%)	14 (31.8%)	<0.0001
Post-op Stroke	124	120 (1.6%)	4 (9.1%)	<0.0001
Post-op Dialysis	31	30 (0.4%)	1 (2.3%)	0.0487
Post-Op Reop for Valvular Dysfunction	16	13 (0.2%)	3 (6.8%)	<0.0001
Post-Op Unplanned Coronary Artery Intervention	24	20 (0.3%)	4 (9.1%)	<0.0001
Post-Op Pacemaker	328	327 (4.3%)	1 (2.3%)	0.5160

**Table 5:** Logistic regression of adverse outcomes.

Variables	Adjusted Odds Ratio	95% Confidence Interval	*p* Value
Conversion of Operative Approach	21.71	9.78–48.2	**<0.0001**
Aortic Dissection	28.13	6.16–128.51	**<0.0001**
Vascular Complications	8.31	4.06–16.99	**<0.0001**
Cardiac Arrest	55.67	29.27–105.9	**<0.0001**
Bleeding	19.47	10.15–37.35	**<0.0001**
Post-op Stroke	6.31	2.22–17.91	**0.0005**
Post-op Dialysis	5.94	0.79–44.54	0.0831
Post-Op Reop for Valvular Dysfunction	43.22	11.87–157.37	**<0.0001**
Post-Op Unplanned Coronary Artery Intervention	38.37	12.55–117.28	**<0.0001**
Post-Op Pacemaker	0.52	0.07–3.82	0.5232

## Data Availability

The datasets presented in this article are not readily available due to membership limitations within the statewide collaborative. Please reach out to Eddie Fonner at the email address eddie@vcsqi.org for access to the data.

## Abbreviations

The following abbreviations are used in this manuscript:

aOR	adjusted odds ratio
CI	confidence interval
ECMO	extracorporeal membrane oxygenation
IABP	intra-aortic balloon pump
MI	myocardial infarction
NYHA	New York Heart Association
pVAD	percutaneous ventricular assist devices
SAVR	surgical aortic valve replacement
SD	standard deviation
TAVR	trans-catheter aortic valve replacement
tMCS	temporary mechanical circulatory support

## References

[1] Liang Y., Dhoble A., Pakanati A., Zhao Y., Kork F., Ruan W., Markham T., Smalling R., Balan P., Estrera A. (2021). Catastrophic Cardiac Events During Transcatheter Aortic Valve Replacement. Can. J. Cardiol..

[2] Almajed M.R., Mahmood S., Obri M., Nona P., Gonzalez P.E., Chiang M., Wang D.D., Frisoli T., Lee J., Basir M. (2023). Application of Impella Mechanical Circulatory Support Devices in Transcatheter Aortic Valve Replacement and Balloon Aortic Valvuloplasty: A Single-Center Experience. Cardiovasc. Revasc. Med..

[3] Banga A., Bansal V., Pattnaik H., Amal T., Agarwal A., Guru P.K. (2024). Extracorporeal Membrane Oxygenation-Supported Patient Outcome Undergoing Transcatheter Aortic Valve Replacement. ASAIO J..

[4] Shou B.L., Verma A., Florissi I.S., Schena S., Benharash P., Choi C.W. (2022). Temporary Mechanical Circulatory Support for Transcatheter Aortic Valve Replacement. J. Surg. Res..

[5] Iantorno M., Ben-Dor I., Rogers T., Gajanana D., Attaran S., Buchanan K.D., Satler L.F., Shults C.C., Thourani V.H., Waksman R. (2018). Emergent valve-in-valve transcatheter aortic valve replacement in patient with acute aortic regurgitation and cardiogenic shock with preoperative extracorporeal membrane oxygenator: A case report and review of the literature. Cardiovasc. Revasc. Med..

[6] Elixhauser A., Steiner C., Harris D.R., Coffey R.M. (1998). Comorbidity measures for use with administrative data. Med. Care.

[7] Nashef S.A.M., Roques F., Sharples L.D., Nilsson J., Smith C., Goldstone A.R., Lockowandt U. (2012). EuroSCORE II. Eur. J. Cardiothorac. Surg..

[8] Kim H.N., Yang D.H., Park B.E. (2023). Acute decompensated heart failure after transcatheter aortic valve implantation: A case report. Clin. Case Rep..

[9] Takahashi Y., Toba T., Otake H., Kawamori H., Tanaka H., Hirata K. (2022). Myocardial Stunning with Severe Functional Mitral Regurgitation in Transcatheter Aortic Valve Replacement―Temporal Change in Transesophageal Echocardiographic Findings. Circ. Rep..

[10] Fabbro M., Goldhammer J., Augoustides J.G.T., Patel P.A., Frogel J., Ianchulev S., Cobey F.C. (2016). CASE 1—2016 Problem-Solving in Transcatheter Aortic Valve Replacement: Cardiovascular Collapse, Myocardial Stunning, and Mitral Regurgitation. J. Cardiothorac. Vasc. Anesth..

[11] Mengi S., Urena M., Veiga-Fernandez G., Alperi A., Nombela-Franco L., Vilalta V., Regueiro A., Mesnier J., Fradejas-Sastre V., Avanzas P. (2025). Impact of Prior Q-Wave Myocardial Infarction in Transcatheter Aortic Valve Replacement Patients with Reduced Ejection Fraction. Struct. Heart.

[12] Pibarot P., Dumesnil J.G. (2012). Low-flow, low-gradient aortic stenosis with normal and depressed left ventricular ejection fraction. J. Am. Coll. Cardiol..

[13] Miller P.E., Senman B.C., Gage A., Carnicelli A.P., Jacobs M., Rali A.S., Senussi M.H., Bhatt A.S., Hollenberg S.M., Kini A. (2024). Acute Decompensated Valvular Disease in the Intensive Care Unit. JACC Adv..

[14] Seco M., Forrest P., Jackson S.A., Martinez G., Andvik S., Bannon P.G., Ng M., Fraser J.F., Wilson M.K., Vallely M.P. (2014). Extracorporeal Membrane Oxygenation for Very High-Risk Transcatheter Aortic Valve Implantation. Heart Lung Circ..

[15] Tribouilloy C., Rusinaru D., Maréchaux S., Castel A.-L., Debry N., Maizel J., Mentaverri R., Kamel S., Slama M., Lévy F. (2015). Low-Gradient, Low-Flow Severe Aortic Stenosis with Preserved Left Ventricular Ejection Fraction. J. Am. Coll. Cardiol..

[16] Wagener M., Reuthebuch O., Heg D., Tüller D., Ferrari E., Grünenfelder J., Huber C., Moarof I., Muller O., Nietlispach F. (2023). Clinical Outcomes in High-Gradient, Classical Low-Flow, Low-Gradient, and Paradoxical Low-Flow, Low-Gradient Aortic Stenosis After Transcatheter Aortic Valve Implantation: A Report from the SwissTAVI Registry. J. Am. Heart Assoc..

[17] Prakash Y., Chopra L., Mannina C., Galvani E., Akinmolayemi O., Singh R., Argulian E., Melarcode-Krishnamoorthy P., Dangas G., Halperin J.L. (2025). Comparative Outcomes of Transcatheter Aortic Valve Replacement and Conservative Management in Patients with Low-Flow, Low-Gradient Aortic Stenosis. Am. J. Cardiol..

[18] Delgado V., Clavel M.-A., Hahn R.T., Gillam L., Bax J., Sengupta P.P., Pibarot P. (2019). How Do We Reconcile Echocardiography, Computed Tomography, and Hybrid Imaging in Assessing Discordant Grading of Aortic Stenosis Severity?. JACC Cardiovasc. Imaging.

[19] Bhogal S., Rogers T., Aladin A., Ben-Dor I., Cohen J.E., Shults C.C., Wermers J.P., Weissman G., Satler L.F., Reardon M.J. (2023). TAVR in 2023: Who Should Not Get It?. Am. J. Cardiol..

[20] Weich H.S.V., John T.-J., Joubert L., Moses J., Herbst P., Doubell A. (2021). Dynamic Left Ventricular Outflow Tract Obstruction Post–Transcatheter Aortic Valve Replacement. JACC Case Rep..

[21] Joseph L., Bashir M., Xiang Q., Yerokun B.A., Matsouaka R.A., Vemulapalli S., Kapadia S., Cigarroa J.E., Zahr F. (2018). Prevalence and Outcomes of Mitral Stenosis in Patients Undergoing Transcatheter Aortic Valve Replacement?. JACC Cardiovasc. Interv..

[22] Tamburino C., Capodanno D., Ramondo A., Petronio A.S., Ettori F., Santoro G., Klugmann S., Bedogni F., Maisano F., Marzocchi A. (2011). Incidence and Predictors of Early and Late Mortality After Transcatheter Aortic Valve Implantation in 663 Patients with Severe Aortic Stenosis. Circulation.

[23] Singh V., Mendirichaga R., Inglessis-Azuaje I., Palacios I.F., O’Neill W.W. (2018). The Role of Impella for Hemodynamic Support in Patients with Aortic Stenosis. Curr. Treat. Options Cardiovasc. Med..

[24] Martinez C.A., Singh V., Heldman A.W., O’Neill W.W. (2013). Emergent use of retrograde left ventricular support in patients after transcatheter aortic valve replacement. Catheter. Cardiovasc. Interv..

[25] Radsel P., Goslar T., Bunc M., Ksela J., Gorjup V., Noc M. (2021). Emergency veno-arterial extracorporeal membrane oxygenation (VA ECMO)-supported percutaneous interventions in refractory cardiac arrest and profound cardiogenic shock. Resuscitation.

[26] Lorusso R., Maria Raffa G., Alenizy K., Sluijpers N., Makhoul M., Brodie D., McMullan M., Wang I.-W., Meani P., MacLaren G. (2019). Structured review of post-cardiotomy extracorporeal membrane oxygenation: Part 1—Adult patients. J. Heart Lung Transplant..

[27] Ayala S., Ma Z., Peng K., Ji F., Li D. (2024). Postoperative Acute Kidney Injury After Transcatheter Aortic Valve Replacement. Curr. Anesthesiol. Rep..

[28] Almarzooq Z.I., Kazi D.S., Wang Y., Chung M., Tian W., Strom J.B., Baron S.J., Yeh R.W. (2022). Outcomes of stroke events during transcatheter aortic valve implantation. EuroIntervention.

[29] Ferruzzi G.J., Silverio A., Giordano A., Corcione N., Bellino M., Attisano T., Baldi C., Morello A., Biondi-Zoccai G., Citro R. (2023). Prognostic Impact of Mitral Regurgitation Before and After Transcatheter Aortic Valve Replacement in Patients with Severe Low-Flow, Low-Gradient Aortic Stenosis. J. Am. Heart Assoc..

[30] Ali J.M., Abu-Omar Y. (2020). Complications associated with mechanical circulatory support. Ann. Transl. Med..

